# LoRaWAN Physical Layer-Based Attacks and Countermeasures, A Review

**DOI:** 10.3390/s22093127

**Published:** 2022-04-19

**Authors:** Henri Ruotsalainen, Guanxiong Shen, Junqing Zhang, Radek Fujdiak

**Affiliations:** 1 Institute of IT Security Research, St. Pölten University of Applied Sciences, Campus-Platz 1, 3100 St. Pölten, Austria; 2 Department of Electrical Engineering and Electronics, University of Liverpool, Liverpool L69 3GJ, UK; guanxiong.shen@liverpool.ac.uk (G.S.); junqing.zhang@liverpool.ac.uk (J.Z.); 3 Department of Telecommunications, Brno University of Technology, Technicka 12, 61600 Brno, Czech Republic; fujdiak@vutbr.cz

**Keywords:** LoRaWAN, vulnerabilities, security, physical layer, hardware security

## Abstract

As LoRaWAN is one of the most popular long-range wireless protocols among low-power IoT applications, more and more focus is shifting towards security. In particular, physical layer topics become relevant to improve the security of LoRaWAN nodes, which are often limited in terms of computational power and communication resources. To this end, e.g., detection methods for wireless attacks improve the integrity and robustness of LoRaWAN access. Further, wireless physical layer techniques have potential to enhance key refreshment and device authentication. In this work, we aim to provide a comprehensive review of various vulnerabilities, countermeasures and security enhancing features concerning the LoRaWAN physical layer. Afterwards, we discuss the impact of the reviewed topics on LoRaWAN security and, subsequently, we identify research gaps as well as promising future research directions.

## 1. Introduction

### 1.1. Motivation

Within the last six years, LoRaWAN has evolved from its first specification (v1.0, published in 2015) to a strong contender among low-power wide-area network (LPWAN) technologies. During this time frame, LoRaWAN technology has boosted connectivity for many applications, e.g., in agriculture, logistics, smart lighting and waste management. Furthermore, the popularity and the availability of the underlying LoRa modulation technology has initiated further development in research areas such as battery free back-scatter communications [1], open-source network components, e.g., the chirpstack [2], and enhanced medium-access strategies with increased throughput [3]. Recently, satellite-based LoRaWAN communication links have also been demonstrated [4], which aim to pave the way for operation without gateways, which is especially well-suited for LoRaWAN employment in rural areas.

Despite the advances and rapidly gained popularity, the state of security in LoRaWAN networks is still inherently imbalanced. On the backend side, the servers and their interconnections can be flexibly secured and updated with the latest available software components, such as public key infrastructure, virtual private network connections, secure protocols, intrusion detection systems and firewalls [5]. Therefore, the frontend side of the network, consisting of lightweight embedded nodes, typically implements only a fragment of the available security features. Although the LoRaWAN specification gives definitions for, e.g., authenticated network join procedures and session key derivations [6], further secure development steps are left for the user to implement. Further obstacles might arise due to dissimilar attack surfaces on the backend and on the frontend. For instance, distributed denial-of-service (DoS) and its countermeasures are well understood network security concepts. However, DoS attacks targeted at LoRaWAN nodes, e.g., via reactive RF jamming, are much harder to thwart. Finally, large scale employments of LoRaWAN with hundreds of nodes might pose security management problems on the application layer, given that a procedure for over-the-air updates for firmware patching is not defined in the current LoRaWAN specification.

### 1.2. LoRaWAN Security

One of the major development goals of LoRaWAN is to lift the level of frontend security to be on-par with the level of its backend security. To this end, several efforts have been made to expose attack vectors in the protocol and at the LoRaWAN device level. Shortly after the publication of the LoRaWAN v1.0 specification, security analysts were able to identify vulnerabilities in the LoRaWAN medium-access protocol. For example, a bit-flipping attack [7] allows an attacker to issue malicious modifications to an encrypted payload, which enables tampering with, e.g., sensor values. Further analysis [8] indicated shortcomings in replay attack protection for the network join procedure and the downlink acknowledgement messages. These network layer vulnerabilities have been reviewed earlier, in [9], where a more comprehensive description of the attacks can be found. More recently, security issues on the physical layer have been reported as well. For instance, by launching DoS attacks against LoRaWAN nodes, the device’s current consumption is drastically increased, which leads to battery depletion. This kind of denial-of-sleep attack [10] hse been reported for LoRaWAN, e.g., in [11]. On the wireless physical layer, proof of concepts for triggered- and reactive-jamming attacks have been given [12], which also play a significant role in wormhole attacks [13].

Next to studies involving vulnerabilities in LoRaWAN, research efforts have been devoted to system hardening and to the development of novel protection mechanisms on the physical layer, such as device authentication based on LoRa transmitter fingerprinting [14,15,16,17,18] and wireless secret key agreement [19,20,21]. The core idea behind protection techniques on the physical layer is to make use of existing physical components as building blocks to improve security and trust. For instance, key agreement techniques can be utilized to refresh root keys to enable secure communication. To this end, LoRaWAN is a well-suited target for an approach such as the security functions of the protocol, i.e., encryption and message integrity checks, that rely heavily on symmetric key cryptography. Further protection topics involve jamming-detection [22,23] and jamming-prevention [24] mechanisms. Since LoRaWAN is freely accessible and the monitoring of the network operation for security incidents is left as the responsibility of the user, it is highly relevant to study ways to improve the resilience of the wireless communication in LoRaWAN networks. 

### 1.3. Related Work

Physical-layer-based security techniques have been widely studied for wireless technologies, including WiFi, ZigBee, Bluetooth. These techniques can be mainly categorized into key generation and device authentication [25]. Key generation exploits the unique and reciprocal characteristics of wireless channels as cryptographic keys. A comprehensive review has been given in [26] and it has been widely studied for WiFi [27], ZigBee [28] and Bluetooth [29]. Physical-layer-based device authentication relies on unique and stable hardware impairments and exploits their features as device identifiers [30]. Any wireless devices have such impairments, which result from the manufacturing process. Similar to key generation, there have been many research efforts on leveraging hardware impairments as fingerprints for WiFi [31] ZigBee [32], and Bluetooth [33].

During the past few years, several review papers have been published on LoRaWAN security, of which many concentrate on basic security features and attack/defense methods. For instance, in [9], the authors identify potential threats and countermeasures and deliver recommendations for system hardening. In a similar vein, ref. [34] focused on applied LoRaWAN security, where attacks and protection methods were identified from the perspective of a real-world agriculture use case. Further, the authors of [35] compared security features and issues between the LoRaWAN specifications 1.0 and 1.1, and subsequently highlighted security needs for specification 1.1. The works aiming at more systematic threat analysis include, firstly, [36], where an analysis on confidentiality, availability and integrity along with relevant state-of-the-art works was given. Secondly, ref. [37] presented a comprehensive threat catalogue for LoRaWAN specification 1.1, which becomes useful while, e.g., performing risk analysis of a LoRaWAN use case. Finally, a review of authentication methods concentrating on light-weight techniques suitable for LoRaWAN devices was presented in [38].

### 1.4. Novelty and Structure

As reported in the previous section, the state-of-the-art literature includes review/survey papers with references to vulnerabilities and countermeasures on different communication layers. Motivated by the scope of this paper, we reviewed the contents of the surveys [9,34,35,36,37,38] regarding their presentation of physical layer security topics. As presented in Table 1, only works [9,34,37] delivered brief reviews on the relevant physical-layer attacks and mentioned some countermeasures. Nevertheless, none of these works treated these topics comprehensively and they also lacked discussion on wireless physical-layer techniques. Therefore, it is of our interest to bridge this gap by presenting a consistent review of the relevant physical-layer vulnerabilities and protection topics. For this review, we have included articles from the major publishers (e.g., IEEE/ACM/Elsevier/MDPI), which fulfill two main criteria: (1) the method/application involves LoRaWAN as a means of communication, and (2) the security topic of the paper is strictly connected to the physical layer (including both the device layer and wireless physical layer).

The rest of the paper is organized as follows. After giving a brief introduction to the key components of LoRaWAN in Section 2, we move on to present the various physical layer vulnerabilities in Section 3. Subsequently, the protection topics are given in Section 4, after which we aim to deliver discussion points on LoRaWAN security and further research steps in Section 5. Ultimately, we conclude the paper in Section 6.

## 2. LoRaWAN

This section covers different aspects of LoRaWAN: the physical layer, medium access, the network structure, the packet structure and security features. The given background becomes useful while studying vulnerabilities and protection techniques in the later sections.

### 2.1. Network Topology

**Figure 1:**
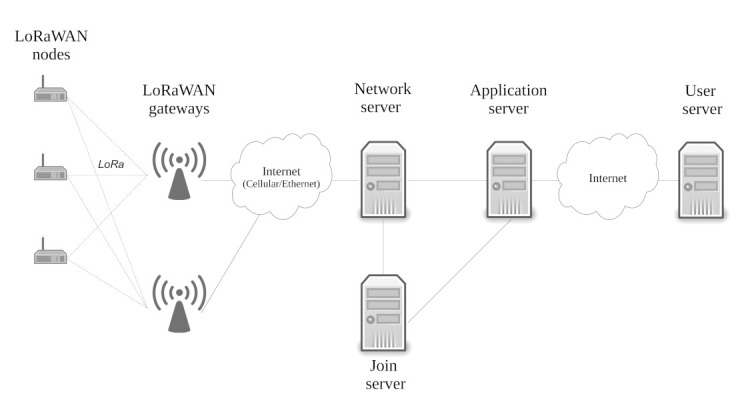
Overview of the LoRaWAN network architecture.

A LoRaWAN network consists of the following fundamental elements, as illustrated in Figure 1: nodes, gateways, a network server, a join server and an application server. The structure of a LoRaWAN network follows the star-of-stars topology, where nodes are connected towards a central network server and an application server via multiple gateways. Such settings can improve data throughput, as variable RF channel conditions might temporarily become degraded on a single gateway.

A LoRaWAN node initiates the network join procedure as well as taking care of secure data transfer towards the servers. In between the server infrastructure and the nodes, several gateways forward LoRaWAN data packets and MAC layer commands. During LoRaWAN packet reception, a gateway decides between forwarding and packet drop by inspecting the integrity of the received packet. Next to the LoRaWAN payload, additional information such as a time stamp or metadata, such as a received signal strength indicator (RSSI) value or a signal to noise ratio (SNR) value, are added to the forwarded message. The link between a gateway and the servers can be implemented by any available communication infrastructure (WLAN, 4G/5G, Ethernet). The network server (NS) handles all the essential features on the network level, such as the frame authentication, responding to MAC layer requests, the gateway selection for downlink messages and the data rate adaptation for the nodes. The gateway selection is based on the ranking of the recorded RSSI/SNR. Additionally, as a single LoRaWAN payload might arrive multiple times from many gateways, the NS performs deduplication of the messages. The join server takes care of the over-the-air activation procedure, during which a node is authenticated and the sessions keys are derived. Finally, the application server is utilized to decrypt the LoRaWAN messages and to store the payload data (and the metadata).

### 2.2. Medium Access

The LoRaWAN specification defines three modes of MAC layer operation, namely, Classes A, B, and C. Depending on the needs of a LoRaWAN application, the mode for a low-power operation (Class-A), for a lower latency (Class-B) or for a high data availability (Class-C) can be selected.

**Figure 2:**
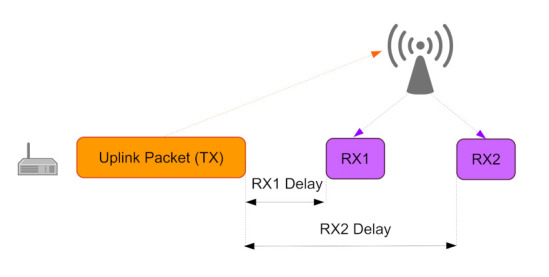
LoRaWAN reception windows in Class-A operation mode.

In the Class-A operating mode, LoRaWAN nodes utilize the simple pure ALOHA medium access method. Hence, the nodes transmit their payloads, in a scheduled or in an event-based manner, towards a gateway without channel-activity sensing. The gateway can, in turn, communicate towards the nodes via downlink connection. The downlink packets include mostly the acknowledgement packets utilized for confirmed messaging and the MAC commands. For the downlink data, as illustrated in Figure 2, two reception time slots on the node, often referred to as RX1 and RX2, are activated in Class-A mode. The reception times are scheduled 1 s or 2 s after the transmission of an uplink LoRaWAN payload has been finished. This way, LoRaWAN nodes can spare energy, as the microcontroller and the LoRa modem of a node can enter sleep mode between uplink packets and between the transmission and the reception windows.

The LoRaWAN Class-B operation mode is devoted to higher data availability. As an extension to Class-A, Class-B introduces periodical reception time windows, which can be helpful to implement long-range applications, where a continuous bidirectional data transfer is required. Nodes synchronize themselves to the broadcast synchronization beacon message.

Class-C extends data availability beyond the options given by Class-A and Class-B. With an unlimited reception time window, nodes can immediately receive downlink packets, which is beneficial in terms of low-latency applications. As nodes are required to be activated periodically for receptions in Class-B/C modes, the energy consumption is higher as activation of sleep modes become more sparse. Thus, these modes are better suited for mains-powered, always-on applications, such as smart meters.

Next to data packets, the LoRaWAN specification defines several MAC command packets that are delivered bidirectionally between a node and a gateway. These commands can be utilized to, e.g., set the maximum aggregated transmit duty cycle of the node, inquire the status of the node and request to change downlink reception frequency. The complete list of MAC commands can be found in [6].

### 2.3. LoRa Physical Layer

**Figure 3:**
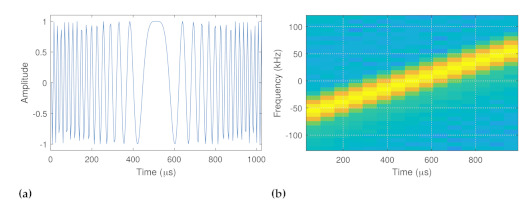
(**a**) I−branch of the LoRa signal. (**b**) Spectrogram of the LoRa signal.

Physical-layer signaling, which is used to carry LoRaWAN payloads, is defined by the proprietary LoRa chirp-spread-spectrum (CSS) modulation, whose frequency increases linearly. The I branch of the LoRa preamble and its spectrogram are given in Figure 3.

As the LoRa modulation format is designed with robustness against noise and interference in mind, LoRa packets can be correctly received even for signal-to-noise ratios (SNR) well below zero. Hence, a wide communication range both indoors and outdoors can be achieved with LoRaWAN. During LoRa modulation, the MAC-layer payload bits are converted into several chirp signals, where the different bit strings of a payload are conveyed to the physical-RF signal by phase modulation. The capacity of a chirp in LoRa is defined by the so called spreading factor (SF), which can take discrete values from 7 to 12. Hence, a single chirp can hold, at most, SF bits. Since, in LoRa, the signal bandwidth and the phase resolution are fixed, a change in SF directly affects the duration of the modulated RF waveform as given by $T=2^{\mathrm{SF}}/\mathrm{BW}$, where BW denotes the bandwidth of a LoRa signal. For instance, LoRaWAN packets of a 16 bytes payload and BW=125kHz with SF=7 or SF=12 translate into LoRa signals with periods of 66 ms and 1646 ms, respectively. Hence, the LoRaWAN data rates and the LoRaWAN device air times are often quite restricted. Nevertheless, LoRa modulation allows for the modulation and demodulation of RF waveforms with moderate complexity, which is favourable from an IoT-application point of view.

Apart from signaling, other physical layer parameters such as center frequency, duty cycling or transmission power are often regionally defined. Since LoRaWAN operation is intended to be free of charge, the typical bands include the licence-free ISM channels, i.e., EU433, EU863-870, AU915-928/AS923-1, US902-928 and IN865-867. Next to channel regulations, regional restrictions to the LoRaWAN packet air time apply. For instance, in the EU, according to the ETSI EN300.220 standard, the devices must comply with duty cycles between 0.1% and 10% depending on the used sub-band.

RF transceivers with built-in support for LoRa include those from Semtech. On LoRaWAN devices, the popular SX1276-79 modules are favoured, which offer a wide tuning range from 137 MHz up to 1020 MHz, a maximum RF transmission power of 20dBm and a maximum link budget of 168dBm. Next to LoRa waveform modulation and demodulation, a transceiver integrates a direct conversion transmitter and a direct conversion receiver in a single chip package. From an application point of view, e.g., a low-power microcontroller-based wireless sensor, a LoRa transceiver offers useful features such as so called wake-up interrupts, which notify the host after a successful detection of a preamble, detection of a sync address or decoding of a payload. As it turns out, such features are also useful for implementing reactive jamming on a LoRa hardware. On the LoRaWAN gateway side, Semtechs SX130x digital baseband chips are utilized. They include a demodulation path, which is able to demodulate signals on multiple neighboring subchannels and consequently decode several LoRa packets in parallel. Typically, the baseband modems are accompanied by the SX125x analog frontends, which take care of RF signal up/down conversions as well as digital-to-analog and analog-to-digital conversions.

For more detailed descriptions of the LoRa modulation and the related RF components, we refer an interested reader to, e.g., [39].

### 2.4. LoRaWAN Packet Structure

**Figure 4:**
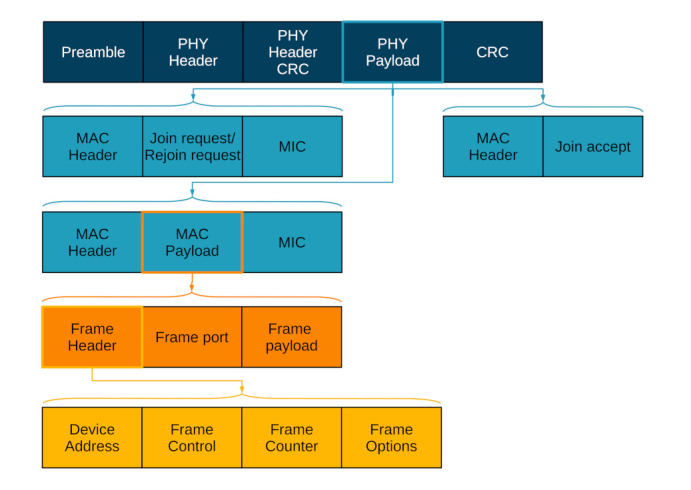
Overview of the LoRaWAN packet structure.

Figure 4 delivers an overview of the messages involved in LoRaWAN [6]. Each LoRaWAN payload is contained in a physical-layer LoRa packet, which includes a preamble, a physical-layer header and a CRC code. These fields are necessary for LoRa-receiver synchronization, LoRa-receiver demodulation parameters (e.g., coding rate setting) and CRC-error correction. The LoRaWAN payload itself (PHY payload) conveys the MAC message, which, in turn, holds the MAC-payload, join-request, rejoin-request or join-accept parts. The MAC payload contains the frame header, frame port and the frame payload. The first two fields are utilized to, firstly, communicate the MAC commands and, secondly, to carry further important information including the device address and the frame parameters. For the latter, the frame control field specifies, e.g., whether or not the adaptive data rate and/or packet acknowledgement shall be enabled. Further, the frame-counter field contains a counter value, which is utilized to keep track of the number of packets exchanged between an end device and a gateway. The frame-options field is an optional data field carrying the MAC commands. Finally, the frame-payload field carries LoRaWAN application data.

### 2.5. Adaptive Data Rate

With the adaptive-data-rate (ADR) mechanism, a LoRaWAN network is able to optimize the transmission power, air time and data rates of the devices. This optimization step directly translates into a longer device battery lifetime, higher data throughput and also, potentially, into lower latency.

The ADR is based on three main components: a collection of RF-link statistics, MAC commands and an ADR algorithm.

- Collection of RF-link statistics: The algorithm receives the RF link information in SNR values. After a sufficient number of SNR values is collected, a margin to the minimum SNR thresholds of the different spreading-factor settings is calculated. Depending on the margin, the network server can request that a device switch the spreading factor and the transmission power settings accordingly.
- MAC Commands: LoRaWAN defines a few MAC command packets that control the ADR. With the 1-bit **ADR** uplink packet, a device can request that the network server control its data rate. The 1-bit **ADRAckReq** uplink packet is periodically sent by devices to request that the network server validate the received uplink messages. Depending on the network servers response to this packet, the end device can optimize its spreading factor and transmission power to find the optimal communication parameters.
- ADR Algorithm: The 4 bytes long **LinkADRReq** downlink packet contains the parameters determined by the ADR algorithm, which are communicated to the device from the network server. A more comprehensive overview of the algorithm can be found, e.g., from [40].

Typically, ADR is recommended for devices with fixed locations, so that RF-link-quality information can be collected reliably. For mobile devices, additional location sensing (e.g., via GPS localization) is recommended to determine time frames during which the device remains in a fixed location.

### 2.6. Device Activation

The LoRaWAN standards v1.0 and v1.1 define various measures to ensure, e.g., device authentication, message integrity and confidentiality. These fundamental security features are initialized during the so called device activation phase, as the LoRaWAN device joins (or rejoins) the network. In the following, the two ways of device activation, i.e., the over-the-air activation (OTAA) and the activation-by-personalization (ABP), for the LoRaWAN standards v1.0 and v1.1, are reviewed in a compact form.

#### 2.6.1. LoRaWAN v1.0

Prior to the OTAA procedure in LoRaWAN v1.0, the application identifier **AppEUI**, the device identifier **DevEUI** as well as the secret key **AppKey** are stored in an end device and also provisioned to the network in which the device is joining. As the first step, the device composes a join request message with **AppEUI**, **DevEUI**, a nonce value (**DevNonce**) and a message integrity code (MIC), which is calculated with the join-request-message fields and **AppKey**. Once the join request arrives at the network server and the request is permitted, the network server replies with the join-accept message. This message contains a random nonce (**AppNonce**), a network ID (**NetID**), a device address (**DevAddr**), and parameters for physical-layer signaling, followed by a MIC. The join-accept message is encrypted using **AppKey**. Once the join-accept message arrives at the end device, the application session key (**AppSKey**) and the network session key (**NwkSKey**) are derived using **AppNonce** and **Appkey**. Finally, to establish end-to-end encryption, the network server delivers **AppSKey** to the application server.

In contrast to OTAA, in ABP activation, **DevAddr**, **AppSKey** and **NwkSKey** are a priori programmed on the end device, the network server and the application server.

#### 2.6.2. LoRaWAN v1.1

The LoRaWAN standard v1.1 improves communication security by introducing an additional join-server and further session keys. Similar to the above, the first step in OTAA requires storage of the join identifier (**JoinEUI**), **DevEUI**, **AppKey** and an additional secret key, **NwkKey**, to an end device. The latter three are also stored in the join server. As an end device proceeds to start OTAA activation, it creates the join-request packet, including **DevEUI**, **JoinEUI** and **DevNonce**. The integrity of the join-request packet is protected by the MIC, which is calculated using **NwkKey**. Once the network server approves the join request, it locates the join server defined by **JoinEUI** identifier. Next, a **JoinReq** message is sent to the join server, which contains the original join request message and the device-relevant parameters. Subsequently, on the join server, network-session keys (**SNwkSIntKey**, **FNwkSIntKey**, **NwkSEncKey**) and an application session key (**AppSKey**) are generated, which are later utilized to verify the integrity and the confidentiality of the MAC commands and payload data. The session keys and the join-accept message are encrypted with **NwkKey** and incorporated into a **JoinAns** message, which is delivered back to the network server. Given that **JoinAns** is successfully verified by the network server, the join-accept message is forwarded to the end device, which derives the above session keys out of the message. After the session-key exchange, the application server receives the **AppSKey** from the network server upon the first device uplink message and thus, the end-to-end security for an LoRaWAN v1.1 application is established. The ABP activation in version v1.1 follows the lines of version v1.0 and, so, the session keys and the **DevAddr** are preconfigured on end devices as well as on application and network servers.

### 2.7. LoRaWAN Keys

The security features as defined in LoRaWAN standards rely heavily on the above-described secret keys. Firstly, the preprogrammed **AppKey** (and **NwkKey**, as in LoRaWAN v1.1) takes care of the secure authentication of LoRaWAN devices during the join procedure. Secondly, the derived session keys enable end-to-end encryption between an LoRaWAN device and the application server. For the encryption algorithm, the well-known AES-128 is defined. A further feature, which protects the integrity of the LoRaWAN application data, is the frame counter. By synchronizing to the up-link counter values, the network server can detect potential spoofing attempts. Thirdly, the integrity of the LoRaWAN messages is guaranteed with the MIC, which, effectively, is a cryptographic signature calculated with the AES-CMAC algorithm.

## 3. Vulnerabilities

In this section we review relevant vulnerabilities, which involve wireless physical-layer attacks such as sniffing, jamming and wormhole attacks as listed in Table 2. Furthermore, key-extraction attacks as well as energy attacks are reported.

### 3.1. Sniffers

The eavesdropping of wireless communication is one of the most trivial and well-known attacks aiming at wireless protocols including LoRaWAN. The major difference in eavesdropping long-range protocols versus short- to mid-range protocols, is the smaller number of receivers required to acquire communication from a certain area. As indicated by [52], LoRa signaling is robust against fading and interference, which gives an advantage to eavesdropping attempts as well. While the sniffing of LoRa and LoRaWAN payloads is, in its simplest form, possible with a single low-cost LoRa modem, physical-layer sniffers give an attacker a further advantage, as more fine-grained information on communication parameters, e.g., center frequency, becomes available. Below, we feature some of the available tools for signal capture, analysis and decoding that can be applied to LoRaWAN eavesdropping.

**Figure 5:**
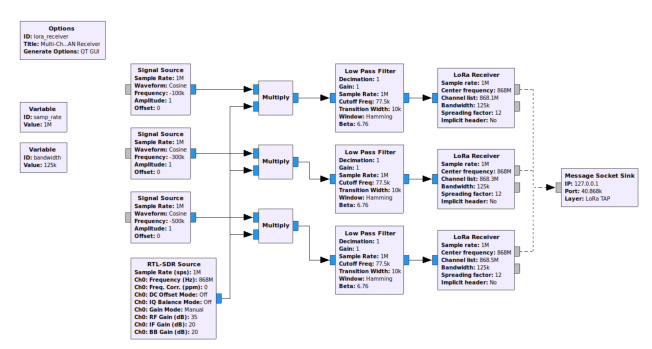
A software-defined multichannel LoRaWAN receiver in GNU Radio.

The state-of-the-art methods to perform physical-layer eavesdropping are mostly based on software defined radio (SDR) hardware and software. As indicated, e.g., by [42], due to narrowband signal characteristics in LoRaWAN, an SDR hardware with moderately low sampling rates, e.g., 1 MSPS, can be utilized for signal capturing. For the subsequent LoRa signal demodulation and decoding stages, a few implementations on the GNU-Radio-SDR-development environment have been presented [53]. In Figure 5, a multichannel LoRaWAN receiver is depicted, which utilizes multiple software-frequency-shifting operations connected to LoRa-receiver blocks in parallel, to extract LoRaWAN payloads. Such a setting allows payload reception on up to 21 channels. As the GNU-Radio-based receivers are open-source, fine-grained information on LoRa waveforms such as carrier frequency offsets, symbol clock periods or received signal strength (RSSI) can be flexibly collected with such receivers.

One of the earliest applications of SDR-based sniffers involved the reverse engineering of LoRa demodulation and decoding, which are proprietary and, thus, not completely available to the public. As discussed in [41], the custom-built LoRa receiver in a GNU radio enabled the reverse engineering of some critical functions of a real-world LoRa decoder, such as data whitening and interleaving, which did not comply with the original LoRa patent. Thus, one can argue that such obfuscation steps, in hopes to, e.g., protect intellectual property, might be circumvented by SDR hardware and the related signal-analysis software. In further works concerning LoRaWAN security, SDR sniffers play a significant role as a basis technology to implement more advanced attacks. For instance, a man-in-the-middle attack test bench with SDR-based sniffers was presented in [43]. Additionally, an SDR LoRaWAN receiver can be applied as a listening unit within a reactive LoRaWAN jammer [24]. Finally, as indicated recently by [44], the flexibility of an SDR is useful to create a versatile security test bed, to assess multiple vulnerabilities, i.e., sniffing, a replay attack, a man-in-the-middle attack and jamming using a single SDR transceiver.

### 3.2. Covert Channels

Covert channels, as an attacker tool, represent a way to transmit sensitive information such as secret keys with a transmission medium, which is often not intended for communication purposes [54]. A classical example includes an optical covert channel, where the intensity of, e.g., an led light is modulated to transmit data. Typical victim devices of covert channels are primarily air-gapped systems and secure-server infrastructure but today, proof-of-concept covert channels with smartphones and IoT gadgets have been presented as well [55]. In the case of LoRaWAN, the authors of [45] presented a hidden communication channel built on top of LoRa signaling. The key component of the method involved embedding-amplitude modulation to a physical LoRa payload. Since LoRa receivers are effectively utilizing frequency demodulation to extract the chirp symbols, an additional amplitude component remains hidden from legitimate users. The authors demonstrated the functionality of the channel with two experimental setups. Firstly, amplitude-modulated LoRa payloads were generated fully in software with gr-lora [53] and transmitted by SDR transmitter. Secondly, amplitude modulation could be achieved with commodity LoRa transceivers by changing the impedance of the transmission line between the LoRa modem and the antenna. The hidden amplitude information can be extracted with an envelope detector and a symbol-recovery mechanism. According to the authors, a covert-communication range of up to 250 m, with approximately 38 bits per a typical LoRa packet, can be achieved by such a signaling technique. Thus, a leakage of **AppKey** (or **AppSKey**) can be devised with only five LoRaWAN packets.

### 3.3. Jamming Attacks

Intentional interference on a wireless physical layer, i.e., jamming, is a well-known DoS attack that has its roots in military communications. Various kinds of jamming attacks have been explored for many wireless protocols [56]. The classical jamming attack involves continuous jamming, where an adversary blocks a wireless channel with a powerful RF signal, which masks legitimate signaling. Recently, more advanced attacks have also been introduced, such as reactive jamming (also known as selective jamming) [56], where an additional listener node is introduced to inform the jammer node on incoming RF payloads. Thus, jamming can be performed against selected devices or signals.

As LoRaWAN networks operate on unlicensed bands, e.g., the EU868 band in Europe, other wireless devices sharing the wireless channel might cause unintentional interference. However, the LoRa chirp signaling is designed to be robust against noise and interference. However, an intentional jamming might bring the SNR at the LoRa receiver below a level under which a packet decoding becomes impossible. Hence, studies have been conducted to reveal the impact of (1) packet collisions [57], (2) unintentional RF interference [58,59], and (3) intentional jamming, with studies considering the impact of jamming on LoRaWAN communication [24,46,47] as well as works devoted to proof-of-concept reactive jammer implementations [12,13,60].

In [24], the authors considered the effect of reactive jamming with Gaussian noise to LoRa-packet reception. According to the empirical study, signal-to-interference-and-noise (SINR) levels close to and above zero did not have a significant adversarial effect on the reception, which confirms the robustness of the CSS modulation. When the jammer noise power is increased to well above the original signal power, e.g., SINR<−6 dB, packet reception rate decreases to near zero. Alternatively, utilizing LoRa chirp signals as jamming waveforms increases jamming success, since, firstly, the LoRa packet reception can be locked to the jamming waveform and, secondly, the interference effect during chirp demodulation is potentially higher. To this end, as noted in [24], the frequency and time alignment of the jamming chirp signals is important. For instance, the effect of nonsynchronized LoRa waveform jamming is comparable to the jamming with Gaussian-noise waveforms. On the other hand, if an attacker synchronizes the jammer transmitter to the victim transmitter, the jamming success improves. According to the experimental results, a transmit power of 15 dBm and upwards is adequate for synchronized waveforms to notably degrade packet-reception rate.

Next to directly jamming LoRaWAN packets, the authors of [48,49] have studied a physical layer vulnerability connected to the join procedure and, particularly, to randomness generation on the SX1276 LoRa modem. As it turns out, the entropy for DevNonce generation is collected based on the continuous RSSI sampling feature of the LoRa receiver. By injecting a suitable jamming waveform to the receiver, the output of the randomness generator can be forced into a constant value. If the procedure is continuously repeated, the network server will drop incoming join requests as nonce reuse is not allowed. According to the authors, jamming can be achieved by a high power jamming signal driving the receiver into saturation or, alternatively, by a fixed-power jamming signal, which holds the RSSI at a constant value. Thus, DevNonce-randomness-manipulation attempts lead to a serious DoS attack, which prevents devices from accessing a LoRaWAN network.

In terms of the jammer hardware utilized to collect experimental results in LoRaWAN jamming, various configurations have been presented. A common option is to adopt a full-duplex SDR transceiver that, due to full reconfigurability, can be flexibly tuned for many kinds of jamming attacks. One such setup, including GNU Radio software, was utilized in [24] to perform synchronized reactive jamming. In order to achieve consistent jammer performance, the authors noted that the inherent latency in the setup should be stabilized by properly configuring the GNU-Radio scheduler. Alternatively, off-the-shelf LoRa transceivers can be also configured to jam selected LoRaWAN packets. In [12], the popular SX1276 transceiver was reconfigured to perform byte-by-byte LoRaWAN packet sniffing and, subsequently, to transmit the LoRa-jamming waveforms. Since SX1276 allows for direct inspection of reception FIFO-buffer contents, the jammer can be triggered once a desired stream of bytes is detected. Hence, the latency between detection of, e.g., victim device address and the transmission of a jamming packet, can also be minimized. In the same vein, the channel activity-detection of LoRa modems can be employed to perform triggered jamming, where jamming waveforms are sent as soon as an arbitrary LoRaWAN communication is detected.

### 3.4. Key Extraction Attacks

The confidentiality of secret information is an essential feature of the cryptographic algorithms involved in LoRaWAN. For instance, the **AppKey** should be stored in a secure manner in order to avoid any key-extraction attempts. However, in the reference implementations of LoRaWAN data transmission with the LoRaMAC libraries [61], the **AppKey** is directly defined in the program code. Hence, via physical access, an attacker might be able to extract **AppKey** with a firmware dump or via a microcontroller debug interface [37]. While secure key storage can be implemented with off-the-shelf microcontrollers, information leakage via side channels might be more difficult to avoid, as discussed below.

In side-channel attacks, an attacker aims to indirectly extract sensitive information from a target device by measuring a physical quantity, which is affected by computation of, e.g., an encryption algorithm. A popular side-channel-measurement method involves the capturing of current-consumption waveforms out of which the information, e.g., secret keys, are extracted. A typical victim device, to this end, is a light-weight microcontroller with a relatively low clock rate (of a few MHz). Hence, as most of the LoRaWAN end devices embed such CPUs, side-channel attacks pose a realistic threat to confidentiality.

The state-of-the-art literature reports a few cases of successful key extraction from a LoRaWAN device. The authors in [50] targeted the AES-CTR algorithm of the original Semtech template LoRaWAN-software implementation [61], to reveal **AppSKey** and **NwkSKey** out of recordings of electromagnetic emanations. The assumption here is that the plaintext payload is available to the attacker, e.g., a digitized sensor value. By applying the correlation power analysis technique, as given in [62], the authors could fully recover **AppSKey** and, partly, also **NwkSKey**. Additionally, the application of digital filtering for noise removal reduced the number of necessary trace captures to ca. 26%. In order to further reduce the number of necessary side-channel-trace captures, the authors of [51] proposed a deep-learning-based classifier to extract the secret keys. According to the experimental results, with a pretrained convolutional neural network model, a full recovery of **AppSKey** in ABP-activation mode becomes feasible with under 100 captured traces.

### 3.5. Wormhole Attacks

The malicious rerouting of wireless packets, i.e., a wormhole attack, is a well-studied topic with its roots in wireless ad hoc networks [63]. In its original form, once a malicious out-of-band route between nodes in a mesh network is established, an attacker can manipulate the routing information and, hence, convince wireless nodes to route traffic towards the malicious link. Wormhole attacks have also been introduced in LoRaWAN networks [12,13], whereby the attack methods and the goal of the attack differ from those presented for mesh networks. With properly timed jamming and the replaying of LoRaWAN packets, an attacker can manipulate the metadata of LoRa frames (e.g., RSSI, SNR and timing) and, thus, affect the stability of a LoRaWAN network.

To the best knowledge of authors, the first practical LoRaWAN wormhole attack was introduced in [12]. Since LoRaWAN does apply direct device-to-gateway links instead of multihop communication, a LoRaWAN wormhole necessitates, firstly, a way to block legitimate packets. To this end, a reactive jammer is placed in the network, preferably close to a gateway. The hardware needed to implement the jammer is the same as reported in Section 3.3. A sniffer node is required to trigger the jammer node and to capture LoRaWAN packets to be replayed later on. This node is essentially a LoRa modem configured for continuous reception on a single frequency. As indicated in [12], the sniffer and the jammer nodes should not be located close to each other, so that the sniffer node is able to receive the desired packets. Such settings can, e.g., emulate certain packet losses, as payloads can be selectively jammed and/or replayed. Thus, with even low-cost equipment, an attacker is able to manipulate the quality of service of LoRaWAN networks.

**Figure 6:**
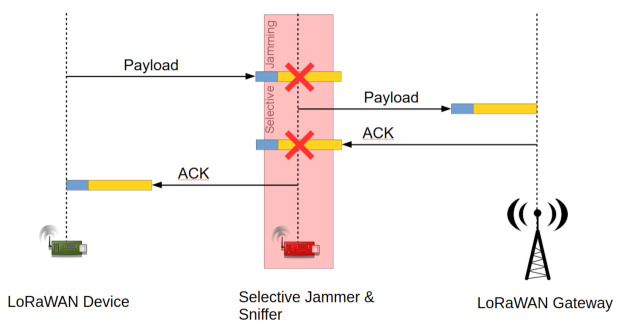
Bidirectional wormhole attack in LoRaWAN.

The wormhole concept was extended in [13], where the authors introduced novel attack concepts based on the methods presented in [12]. One of the architectural differences from the prior work concerned the utilization of an independent reactive-jammer node placed near a gateway. As such, payloads with even the lowest spreading factor can be blocked and replayed later on. With the improved wormhole communication setup, the authors proceeded to discuss the possibility of downlink wormholes. Although applications utilizing LoRaWAN-downlink communication are rare due to channel access restrictions, the uplink wormholes could be detected by missing downlink messages. Thus, by creating a downlink wormhole, as is well-illustrated in Figure 6, an attacker can also target applications where higher data availability is needed. However, as noted by the authors, due to LoRaWAN-reception-window-timing constraints, downlink wormholes can be implemented only for higher data rates and medium-sized payloads within a single payload uplink–downlink transaction. This constraint can be mitigated, given that the downlink message is replayed in a further transaction, which complies with the frame-counter number. Since LoRaWAN wormhole attacks enable manipulation of the metadata (e.g., RSSI or SNR), this feature can be further applied to create DoS and battery-drainage attacks. As an example, given in [13], the ADR mechanism can be manipulated by metadata spoofing. As it turns out, an end device can be forced into spreading-factor and transmission-power settings that cannot be decoded by legitimate packet forwarders.

### 3.6. Energy Attacks

Another class of DoS attacks against battery-powered LoRaWAN devices involves those aiming at maximizing energy consumption [11]. As explained in Section 2.3, for Class-A signaling, the highest energy consumption takes place during packet transmission, during which the RF transmitter and the RF receiver are activated. In order to spare energy, the downlink reception windows are limited in duration and, given that no packets are received during these time windows, the modem is set back to sleep mode. However, if an attacker is able to synchronize to the LoRaWAN-device communication, the LoRa modem can be forced into downlink packet reception mode, which increases the energy consumption significantly. More precisely, an attacker might target the second reception window, for which the reception parameters are typically unchanged from transmission to transmission. Experimental validation of such an attack revealed an increase in energy consumption from 36% to 576%, which, in other words, would reduce a battery-driven device lifetime by several years. As noted in [11], such attacks are more difficult to detect in comparison to, e.g., another kind of energy attack, by forcing a LoRaWAN device to re-transmit packets by direct jamming. Nevertheless, worm-hole attacks in a LoRaWAN network’s supporting-adaptive rate might cause even worse energy consumption, as the attacker is able to force a higher SF setting by manipulating LoRaWAN metadata via replays and packet drops.

## 4. Protection Techniques

In the following, we look at countermeasures, such as detection methods, against replay and jamming attacks. Moreover, radio frequency fingerprinting and wireless key generation methods, beneficial for authentication and key refreshment purposes, are reviewed. A summary of these countermeasures is given in Table 3.

### 4.1. Replay Attack Detection

The state-of-the-art literature considers mostly replay-attack prevention methods pointing to a vulnerability connected to the OTAA procedure, which takes place on a MAC layer. In terms of physical-layer-replay-attacks-protection mechanisms, the authors of [64] introduced a detection technique to an attack vector against gateway-side timestamping, where the delayed forwarding of LoRa waveforms plays the key role. The so called frame-delay attack mechanism closely follows the popular RollJam attack [76], which targets rolling-code-based replay attack prevention in keyless-entry systems. In the LoRaWAN settings, a frame-delay attack necessitates, firstly, an eavesdropper node, which listens for a victim device. Secondly, a collider/replay node takes care of the blocking of the legitimate LoRa frames and replaying of the eavesdropped frames. Hence, arbitrary delays can be introduced to the gateway-side timestamps and, thus, e.g., sensor data arriving late to an application server cannot be distinguished from actual nondelayed data by the commodity LoRaWAN hardware. The authors proposed a physical-layer detection mechanism by extracting frequency bias from LoRa waveforms with an RTL-SDR receiver connected to a LoRaWAN gateway. By comparing known frequency-bias values to collected bias values, replay attempts could be detected even for a signal with an SNR as low as −18 dB.

### 4.2. Wireless Key Generation

**Figure 7:**
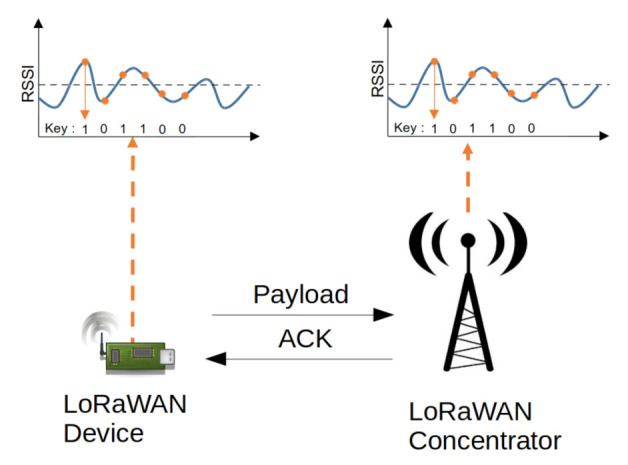
Secret-key-generation in a LoRaWAN network.

The lack of a root-key (i.e., **AppKey** and **NwkKey**) update in LoRaWAN has motivated research towards novel key-generation techniques. As LoRaWAN devices are often based on microcontrollers, it is crucial that added security features do not contribute to a large increase in computational and communication overhead. A potential candidate, which allows for light-weight implementations, is the so called wireless secret-key-generation technique depicted in Figure 7. With its roots in information theory [77], many experimental works on the subject have indicated successful key-agreement results for several wireless protocols [26,78].

**Figure 8:**
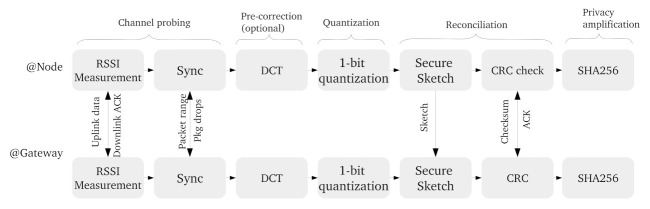
Block diagram of secret-key-generation for LoRaWAN, including necessary communication between a node and a gateway.

One of the basic variants of key generation, illustrated in Figure 8, involves, firstly, a channel-probing phase, i.e., the collection of RSSI values for two wireless devices communicating with each other in a bidirectional manner. Since a wireless channel is reciprocal over a finite time frame, the RSSI readings on both devices are close to each other. Furthermore, due to the randomness and the temporal variations in the channel characteristics, an eavesdropper is unable to arrive at measurements with a similar degree of correlation as the legitimate users. During the second step, a random secret key is extracted out of the RSSI values, e.g., with a simple 1-bit quantization operation. Due to noise and analog component imperfections, the extracted key sequences contain a few bit errors. Those errors can be compensated by so called reconciliation protocols, which allow for error correction without exposing the secret-key material. Finally, privacy amplification techniques should be utilized to minimize key-bit leakage during the error correction phase.

As LoRa communication can be established bidirectionally in LoRaWAN with downlink messages (or, alternatively, in a point-to-point fashion with LoRa packets), RSSI-based key generation becomes an option for LoRa-based key generation. One of the first works to demonstrate experimental results for point-to-point LoRa link was [65], which considered several communication scenarios. It is revealed that a larger SF leads to increased bit errors in extracted keys, which most likely stems from an increased channel measurement delay. Thus, it is challenging to implement LoRa-based key generation, e.g., in mobile scenarios with rapidly changing channel fading conditions. Another work [19] on LoRa-based key generation indicated the importance of differential quantization in channel measurements, with dynamics stemming from path loss and fading. With a suitable key extraction algorithm, the correlation between eavesdropper and the legitimate user(s) can be reduced.

Typically wireless key generation necessitates a fast bidirectional message exchange to avoid imbalance in channel-state estimation. While pure LoRa communication with a low SF can achieve this, the delays in LoRaWAN downlink windows cause latency in channel probing, which potentially gives rise to key bit errors. Furthermore, static channel conditions might result in keys with low entropy, which increases the risk of key-guessing attacks. A potential solution involves randomness injection via a reconfigurable antenna in combination with a novel RSSI preselection algorithm, as presented in [21], which achieves key generation for LoRaWAN communication up to 7 km distance. With such a setting, **AppKey** can be refreshed approximately once per month despite the EU868 channel duty cycle limitations, challenging RF channel conditions (SNR < 0) and the assumed strong eavesdropped attacker model. Additionally, with the RSSI preselection algorithm, the key disagreement rate can be suppressed from 29% down to 18%.

In further works, the challenges connected to LoRaWAN-key generation have been tackled with an optimized key-generation algorithm, as reported in [66,67,68]. For instance [66] presents a multi-bit key-agreement scheme for LoRaWAN, which is optimized for low correlation channel conditions and is thus suitable for mobile and static scenarios. According to extensive experimental results evaluated for static and mobile wireless scenarios, even for correlation coefficient values between 20–60% tolerable KDR values between 10–20% can be achieved. Further, in [67], the authors investigate simple tolerance- and difference-based quantization algorithms and their impact on LoRaWAN key generation. The main result indicated that key refreshment is realizable for LoRaWAN communication in 30 min, given the error-correction capacity of the reconciliation step. Finally, in [68], the authors proposed RSSI precorrection schemes based, firstly, on a discrete cosine transform and, secondly, on principal component analysis. With a blockwise preprocessing, the authors were able to suppress KDR down to a few percent, which indicates the efficiency of such methods in LoRaWAN scenarios where RSSI sets contain noise or nonreciprocity.

### 4.3. Resilience against Jamming

One of the key features of LoRaWAN is the robustness against noise and interference, which allows for higher data availability in difficult signal propagation conditions. However, this condition holds only given that the nature of the interference is unintentional, e.g., another (non-LoRaWAN) device signaling on the same RF channel. Conversely, an intentional RF jamming, as discussed in Section 3.3, might cause a serious DoS attack condition as, potentially, access from hundreds of LoRaWAN devices is blocked. Moreover, since LoRaWAN operates on unlicensed bands such the physical layer, DoS attempts might be more difficult to detect in comparison to conventional DoS attacks in IP networks. Hence, research efforts have been devoted to at least three fronts to measure and to improve resilience of LoRaWAN under jamming attacks.

- Performance evaluations have been conducted to reveal the network system performance under jamming attacks;
- Detection techniques have been developed to distinguish jamming attacks from e.g., faulty gateway/device operation;
- Improved signaling strategies have been presented, which can improve data throughput in the presence of intentional jamming.

In terms of performance evaluations, works studying the impact of jamming have been presented on the physical layer. Firstly, in [69], analytical results were derived for LoRa modulation by considering reactive-band jamming, i.e., noise signals overlapping with a selected part of the LoRaWAN channel bandwidth, and reactive-tone jamming, i.e., jamming with single-/multitone signals. According to the authors, the effect of band jamming was nearly constant, while comparing symbol-error-rate performance for a full-band jammer and for a partial-band jammer. Further, a negligible performance difference was reported between single-tone and multi-tone jammers. As a conclusion, the authors verified the robustness of LoRa signaling against classical noise- and tone-jamming waveforms. Another work [46] studied the effects of jamming with LoRa waveforms with an experimental setup. With a triggered jammer based on a channel-activity-detection algorithm, the authors reported degraded packet delivery ratios (the ratio between the number of correctly received packets and the number of transmitted packets), given that the jamming-waveform power exceeded the legitimate waveform by 6 dB. Another interesting finding concerned a difference in packet-delivery-ratio behaviour while applying constant and triggered jamming. As indicated by the authors, a triggered jamming leads to a worse packet-delivery ratio with less energy overhead. Next to physical layer studies, jamming resilience has also been explored on medium access level. The authors of [70] considered channel-oblivious-jamming attacks and their impact on LoRaWAN-system performance. Given that downlink acknowledgement messages, including payload re-transmissions, are enabled, a network of up to 500 nodes can achieve a reasonably good message-success probability of ca 80% while being attacked on several uplink channels.

Despite the built-in robustness against jamming in limited attack scenarios, as discussed above, carefully executed DoS with the potential for multiple jammers could reduce LoRaWAN payload delivery significantly. Hence, efforts have also devoted to various jammer-detection methods. For instance, [22] presented a detection mechanism based on recurrent neural networks, where RSSI and inter-arrival time values were utilized as model inputs. The model training and validation data sets in the study included data from a LoRaWAN simulator and from a real world test bed. A comparison between several activation functions revealed a detection accuracy, measured using F1 score, of between 0.9 and 0.98. Moreover, according to the author, the inter-arrival time contributed the most to the detection accuracy. Another jammer detector was introduced in [23], which is intended to reveal nonce-manipulation attempts via jamming. As the network server drops join attempts with an already used nonce value, such a procedure is an efficient way to craft DoS attacks in LoRaWAN. With a statistical method employing Kullback–Leibler divergence to compare a legitimate set of nonces and attacked nonces, the authors were able to achieve up to 98% detection accuracy.

Lastly, one of the most effective ways to improve data availability under jamming attacks is to introduce novel decoding and signaling strategies as countermeasures. Techniques regarding the former category were discussed in [24]. Interestingly, as noted by the authors, the collision recovery algorithms for decoding colliding LoRa packets [3,79] are less well suited to separating legitimate payloads from jamming waveforms, given that the jammer is able to synchronize in both the time and frequency domains. Subsequently, by assuming such an advanced attacker model, the authors presented a LoRa decoder that is able to overcome synchronized jamming attempts. The essential idea to enable recovery is to monitor the signal amplitude components during the FFT-based demodulation phase of LoRa payloads. Since a successful jammer needs to adjust signal power such that the jammer waveform power exceeds the legitimate signal power by at least 6 dB, selection of the correct chirp signal components becomes feasible. It should be noted that such a method works well given that the RSSI of the device does not fluctuate heavily, e.g., due to movement or fading. Alternatively, improved medium-access techniques for LoRaWAN have been given [71,72], which employ cryptographic frequency hopping to improve data availability and scalability simultaneously. As noted in [71], cryptographic-frequency hopping can prevent selective jamming attacks to some extent, as the jammer is not able to predict randomly selected channel center frequencies. In order to avoid more powerful jammers listening to several channels in parallel, the authors proposed a continuous center frequency selection over, e.g., the EU868 ISM band, instead of the regular discrete-channel utilization.

### 4.4. Device Fingerprinting

#### 4.4.1. Overview

Radio frequency fingerprint identification (RFFI) is an authentication technique that identifies a device by analyzing characteristics of a received signal, much like authenticating a person with biometrics. The analog frontend of LoRa end nodes is made of low-cost components. These hardware components are subject to manufacturing variations and usually deviate from the nominal values. The deviations are unique to devices and, therefore, can serve as an identifier. In practice, the RFFI system is equipped at the LoRa gateway, and predicts the LoRa transmitter from which the packet is sent by analyzing the received waveform.

**Figure 9:**
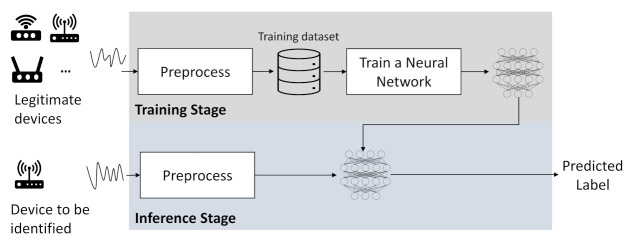
Overview of an RFFI system.

Recently, deep-learning techniques are widely employed in RFFI thanks to their excellent performance in recognition/classification tasks, and LoRa-RFFI systems are mostly deep-learning driven [14,15,16,17,18,73,74,75]. Figure 9 shows the overview of a deep-learning-based RFFI system. A deep-learning-based RFFI system usually has two steps, i.e., training and inference. In the training stage, all the legitimate devices are required to send a number of correctly labelled packets to the RFFI system. The received packets are preprocessed and stored in a training data set. Then, a neural network is trained to learn the mapping function from the received packet to the device label. After sufficient training, the neural network can act as a classifier whose input is the preprocessed LoRa waveform and output is the predicted device label, which is known as the inference stage.

The deep-learning-based LoRa-RFFI works can be categorized into those studying the deep-learning engines, signal representations and channel mitigation. Regarding the deep learning architectures used in LoRa RFFI, a long-short-term memory (LSTM) network [16,18,75], a convolutional-neural network (CNN) [14,15,16,17,18,73,74], multilayer perceptron (MLP) [14,73] and a transformer [80] have been employed. In terms of signal representation, there are studies converting the collected IQ samples into other forms as inputs to the neural network, such as FFT results [14], spectrogram [16,18] and differential constellation trace figure [73]. Finally, the latest research shows that the change in wireless channel degrades the performance of LoRa-RFFI systems [17,18,74]. The authors in [17] designed a channel-independent spectrogram to mitigate channel effects. Moreover, data augmentation is also shown to be helpful [17,18].

#### 4.4.2. Case Study

Twenty commercial off-the-shelf LoPy4 devices and a USRP N210 software-defined radio (SDR) platform are used to experimentally demonstrate how a LoRa-RFFI system works. The carrier frequency of LoPy4 transmitters is set to 868.1 MHz. The transmission is configured with a spreading factor of seven and a bandwidth of 125 kHz. The sampling rate of the USRP N210 receiver is set to 1 MHz. In the experiments, 400 packets are collected in turn from each LoPy4 device and stored in the training dataset, i.e., the dataset contains a total of 8000 packets. The packets are then preprocessed for RFFI, including carrier frequency offset (CFO) compensation, preamble extraction and conversion to channel-independent spectrograms. The CFO compensation is to ensure system stability [15,16], preamble extraction is to prevent learning protocol-specific information and a channel-independent spectrogram is to mitigate the impact of wireless channel [17]. The preprocessed training dataset is then used to train a CNN whose architecture can be found in [17]. After the CNN training stops, we can use it to predict the newly received LoRa packets.

**Figure 10:**
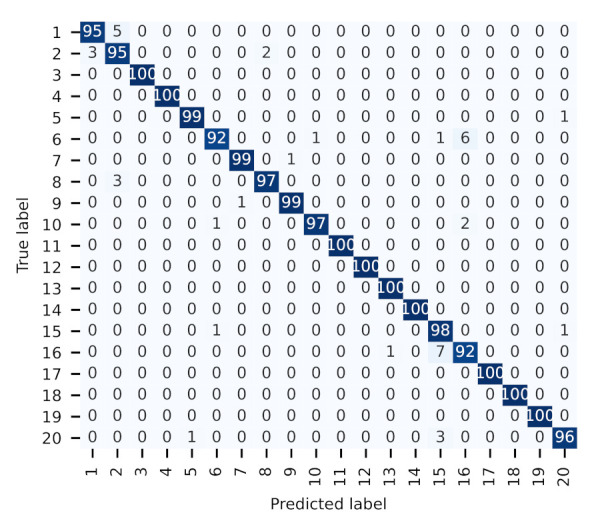
Classification result on LoRaWAN Radio frequency fingerprint identification with the overall accuracy of 97.75%.

After sufficient training, another 100 packets are collected in turn from each LoPy4 device and preprocessed for evaluation/inference. The confusion matrix and overall accuracy are common evaluation metrics for a classification task, which are both presented in Figure 10. As shown in the confusion matrix, most of the predicted labels match the true labels since the predictions distributed along the diagonal, and the overall classification accuracy can reach 97.75%. The result shows that the RFFI system can classify 20 LoPy4 devices with high accuracy, even if they are from the same manufacturer.

## 5. Discussion

In the above sections, we have reviewed several physical-layer attack and protection techniques that are particularly aimed at LoRaWAN networks. Subsequently, in this section, we look forward, to, firstly, analyse the impact on availability, confidentiality and integrity. Secondly, we identify the gaps in the physical-layer security research and, finally, deliver steps for future work.

### 5.1. Lessons Learned—Impact on LoRaWAN Security

#### 5.1.1. Availability

As clearly visible from Table 2 and from Table 3, DoS attacks, in the form of various flavors of jamming and wormhole attacks, have drawn considerable attention. Due to LoRa-chirp-spectrum modulation, a typical LoRaWAN packet airtime spans between milliseconds and seconds. As it turns out, the resulting relatively slow data rate allows for implementations of reactive LoRaWAN jammers with commodity low-cost hardware such as microcontrollers equipped with standard LoRa modems [12]. Consequently, this might enable further threat scenarios for LoRaWAN networks supporting over-the-air updates, as a malicious firmware update could turn a legitimate LoRaWAN device into a jammer. Secondly, the availability of the components to assemble and setup such a jammer does not necessarily require expert level knowledge, which widens the feasibility of LoRaWAN jamming.

In terms of analysing the risk of jamming-based DoS attacks, reactive jamming might result in a higher impact for certain applications. For instance, reactive jamming might pose a serious issue towards applications relying on event-based communication such as flood or gas-leak sensors, since the oblivious jammer can only be first detected after the collision between a jamming frame and a legitimate packet. Hence, such applications would benefit from a so called “heart beat” messages to improve detectability of jamming. Furthermore, as pointed out by [11,13], the blocking of the wireless access is not the only form of DoS in LoRaWAN. Firstly, a LoRaWAN modem can be forced into reception, which increases the current consumption and thus, reduces the device lifetime. Secondly due to the vulnerabilities connected to the adaptive data rate algorithm, the wormhole attacks can force the LoRaWAN device to higher DR parameters, which ultimately causes a large impact on the device battery life.

Parallel to the studies on the attacks themselves, robustness evaluations, as well as novel detection and mitigation strategies, have been demonstrated. For LoRaWAN applications where a packet loss due to a certain degree can be tolerated, the LoRa physical layer is able to support good message throughput even in the presence of a jammer [70]. In such use cases, an additional jamming-detection listener node could be integrated as a part of the intrusion detection system, which delivers information on intentional interference. Lastly, for security-critical applications, the cryptographic frequency hopping medium access techniques offer a viable solution to improve message throughput in the presence of multiple jammers.

#### 5.1.2. Confidentiality and Integrity

The secure connection between a LoRaWAN device and backend servers is heavily dependent on the secure storage and handling of the **AppKey** (and **NwkKey** in LoRaWAN 1.1). In many cases, however, the standard LoRaWAN installations offer only a few measures against physical attacks, which increases the probability of key extraction attacks by means of, e.g., firmware dumps or side-channel attacks. While the former can be thwarted, e.g., using secure elements [81], the latter call for stronger mitigation methods, e.g., tampering-detection mechanisms. A leaked **AppKey** is critical for many reasons. Firstly, it allows for the decryption of the eavesdropped LoRaWAN frames, which decreases communication confidentiality. Secondly, it allows an attacker to mount a rogue end device, which might, e.g., transmit crafted sensor data. Thirdly, a worm-hole attack could be extended towards a more advanced man-in-the-middle attack, where the attacker is able to manipulate both the contents and the metadata of a LoRaWAN packet. These attack vectors pose a serious risk for several LoRaWAN use cases as, via data falsification, the attacker can indirectly affect, e.g., data analysis, visualization or even automation based on LoRaWAN sensing.

As a response to key extraction attacks, wireless-physical-layer techniques have been presented, which can be applied to improve confidentiality and integrity. Wireless secret key agreement techniques provide a low-power alternative to refresh the entire secret key material periodically. Additionally, due to the theoretical information secrecy, wireless key generation is not prone to massive brute-force attacks. This, in turn, is beneficial for the long-term secure storage of LoRaWAN application data. Next, RF fingerprinting methods are a versatile tool for intrusion detection. For instance, with a unique (and unclonable) fingerprint, a legitimate LoRaWAN packet can be distinguished from the one produced by a rogue device, man-in-the-middle attacker or worm-hole attacker.

### 5.2. Research Potential

While several LoRaWAN physical-layer security topics have been comprehensively covered regarding theoretical and experimental research, there is still room for further steps to be taken in order to increase the technical readiness of the existing methods. Furthermore, research potential also lies in, e.g., wireless-physical-layer security topics that have been previously applied to other protocols, but not yet evaluated for LoRaWAN. Hence, in the following, we look forward to reveal potential research directions by proposing relevant follow up research activities. 

#### 5.2.1. Resources

As discussed in Section 2.3, LoRaWAN communication is mostly based on a limited selection of transceiver IC designs from Semtech, namely, the SX127x and the SX130x families. Although this is beneficial in terms of, e.g., compatibility, many details of the Semtech chips remain undisclosed, which is unfavorable when it comes to the realization of wireless-physical-layer security techniques. For instance, the implementations of digital base-band algorithms and, in particular, the parts of it necessary to calculate metadata, e.g., RSSI/SNR, are only partly expressed in the data sheets [82].

A more friendly alternative, to this end, would include a free and open-source hardware implementation of LoRa digital baseband on, e.g., an FPGA. Such a concept already exists for 802.11 based wireless research [83,84], which has alsoexpedited many contributions to physical-layer security. The possibility to define customized signal-processing functions directly on the LoRa transceiver should facilitate, e.g., deployments of more advanced RF-signal sensing and interference cancellation. Furthermore, the user-definable algorithms to perform, e.g., RSSI/SNR estimation, should be useful to enhance the quality as well as the technical readiness of radio-frequency fingerprinting, secret-key agreement and jamming-detection schemes. On the other hand, more advanced reactive jammer-attacker models could be defined with flexible DSP routines. As opposed to the jammer hardware presented with commodity LoRa modems capable of single channel operation only, the advanced reconfigurable hardware supporting wide-band reception will enable preamble detection and reactive jamming on several parallel channels.

Further research focus beneficial for experimental-validation purposes concerns large collections of metadata, i.e., RSSI values or center frequency offsets, from large-scale LoRaWAN networks over longer periods of time. From a resilience point of view, such datasets, including reactive- and triggered-jamming attacks against event-based communication, shall become helpful to determine the accuracy of the presented jamming detectors in real-world conditions. Furthermore, datasets containing metadata from multigateway bidirectional communication with packet collisions are crucial to evaluate the robustness of secret key generation and radio-frequency fingerprinting algorithms. Finally, datasets collected from LoRaWAN use cases, in which attacks on physical layer would cause disasters, e.g., flood/landslide sensors, should accelerate applied research activities around wireless-physical-layer security.

#### 5.2.2. Experimental Research

Many of the works describing vulnerabilities and protection topics mentioned in this paper contain experimental results collected from a small (single node and single gateway) to a middle-scale (5–10 nodes and a single gateway) LoRaWAN network. While such setups deliver acceptable data for proof-of-concept validation, they fail to assess the performance in real operating conditions, i.e., in LoRaWAN networks with hundreds of nodes and several gateways. To this end, further efforts can be made, for instance, towards evaluations for jamming resilience. In particular, a study including several reactive jammers targeting a multi-gateway network will reveal the true effects of physical-layer DoS attacks. Such large scale experiments might also be helpful to study the difference between packet collisions and jamming. In a similar vein, the effectiveness of, e.g., radio-frequency fingerprinting and secret-key agreement techniques should be verified in large scale networks with commodity LoRaWAN equipment. Here, akin to the jamming attack evaluations, a potential focus point might include robustness evaluations against packet collisions during continuous operation.

#### 5.2.3. Further Physical Layer Security Concepts

Wireless-physical-layer security as a research field encompasses several interesting topics, which, to the authors best knowledge, have not been covered before but might be of interest for LoRaWAN security. A good example towards increased resilience and security includes multiple-input-multiple-output (MIMO) antenna techniques, which have already been successfully applied for range extension and angle-of-arrival localization with LoRa waveforms [85]. By using multiple synchronized receivers, as in a MIMO setup, even strong jamming signals can be suppressed without knowledge of the channel characteristics, as demonstrated in [86]. To this end, further development is necessary to apply such methods to LoRaWAN, as most of the state-of-the-art jamming-suppression techniques are based on OFDM signaling instead of the CSS signaling used in LoRa modulation. An additional advantage connected to MIMO techniques concerns randomness generation in wireless secret-key-agreement methods. In particular, opportunistic beamforming, as suggested in [87], can improve key-agreement rate in static wireless-channel conditions. Finally, the MIMO and the beamforming antenna concepts might become helpful to reduce packet collisions in dense LoRaWAN networks.

## 6. Conclusions

In this paper, we reviewed several vulnerabilities and protection methods on the LoRaWAN physical layer. Out of the many published physical-layer topics, the DoS attacks and the related countermeasures, i.e., different flavors of jamming and worm-hole attacks, have received considerable attention. Since the technical feasibility of such vulnerabilities have been demonstrated experimentally with off-the-shelf LoRaWAN hardware, their role in, e.g., the threat assessment of LoRaWAN applications should be carefully taken into account. Jamming detection and prevention techniques have also been presented, to improve the resilience of LoRaWAN communication.

Further important contributions have been carried out to enhance privacy and device authentication. Firstly, several wireless secret-key-agreement methods have been successfully verified in a LoRa/LoRaWAN setting, which have the potential to improve secret-key management in LoRaWAN networks. Secondly, radio-frequency fingerprinting techniques shall become useful tool to verify authenticity of LoRaWAN transmitters. Next, we also analyzed the impact of the physical-layer security topics on LoRaWAN security. Firstly, it became clear that many physical layer attacks, e.g., reactive-jamming or energy attacks, are difficult to thwart/detect with off-the-shelf LoRaWAN equipment alone. Nevertheless, many promising wireless-physical-layer security concepts such, as novel jamming detectors or RF-fingerprinting techniques, are well suited as ingredients for an advanced intrusion detection system aiming at versatile physical layer attack detection. Finally, we recognized the research potential in both applied research and basic research directions. For the former, we identified a need to verify the impact of the vulnerabilities and countermeasures in a large-scale setting. For the latter, we proposed further research towards advanced antenna and/or receiver techniques, which might prove fruitful in terms of privacy and resilience.

## Figures and Tables

**Table 1:** Treatment of physical layer security topics in LoRaWAN security review papers.

Reference	Year	Physical Layer Attacks	Physical Layer Countermeasures
[9]	2020	moderate	moderate
[34]	2021	moderate	moderate
[35]	2020	superficial	-
[37]	2019	moderate	moderate
[36]	2020	moderate	superficial
[38]	2021	-	-
**This work**	2022	**comprehensive**	**comprehensive**

**Table 2:** Overview of reviewed physical-layer vulnerabilities.

Reference	Attack Category	Affects *	Technology	Experimental Results
[41]	Sniffers	C	LoRaWAN 1.0x	yes
[42]	Sniffers	C	LoRaWAN 1.0x	no
[43]	Sniffers	C	LoRaWAN 1.0x	yes
[44]	Sniffers	C	LoRaWAN 1.0x	yes
[45]	Covert Channels	C,I	LoRaWAN 1.0x	yes
[46]	Jamming	A	LoRaWAN 1.0x	yes
[47]	Jamming	A	LoRaWAN 1.0x	no
[12]	Jamming	A	LoRaWAN 1.0x	yes
[13]	Jamming	A	LoRaWAN 1.1/1.0x	yes
[48]	Jamming	A	LoRaWAN 1.0x	no
[49]	Jamming	A	LoRaWAN 1.0x	yes
[50]	Key Extraction	C,I	LoRaWAN 1.0x	yes
[51]	Key Extraction	C,I	LoRaWAN 1.03	yes
[37]	Key Extraction	C,I	LoRaWAN 1.0x	no
[12]	Worm-Hole	A	LoRaWAN 1.0x	yes
[13]	Worm-Hole	A	LoRaWAN 1.1/1.0x	yes
[11]	Energy attack	A	LoRaWAN 1.1/1.0x	yes

* (C—Confidentiality, I—Integrity, A—Availability).

**Table 3:** Overview of physical layer countermeasures.

Ref.	Technique	Enhances *	Advantages	Disadvantages
[64]	Replay detection	C	Comprehensive experimental validation	-
[65]	Secret key agreement	C,I	Experimental validation	No experiments with LoRaWAN
[19]	Secret key agreement	C,I	Quantization for high key randomness	No experiments with LoRaWAN
[21]	Secret key agreement	C,I	Effective over long communication distances	Requires reconfigurable antennas
[66]	Secret key agreement	C,I	Suitable for mobile and stationary nodes	-
[67]	Secret key agreement	C,I	Low algorithmic complexity	Bit disagreement rate
[68]	Secret key agreement	C,I	High secret key entropy	Increased algorithmic complexity
[22]	Jamming detection	A	Versatile modeling tools for performance evaluation	No large scale validation
[23]	Jamming detection	A	High detection accuracy	Only small scale validation
[69]	Jamming resilience	A	-	No experimental validation
[70]	Jamming resilience	A	-	No experimental validation
[24]	Jamming resilience	A	Effective against synchronized jammers	-
[71]	Jamming resilience	A	High performance improvement with low overhead	Acknowledged transmissions not supported
[72]	Jamming resilience	A	High performance improvement	-
[16]	Wireless fingerprinting	U	Investigation on various neural networks	Channel effect is not considered
[73]	Wireless fingerprinting	U	Algorithm for manual extraction of RF fingerprints	Experiments on channel robustness are missing
[74]	Wireless fingerprinting	U	Both indoor and outdoor experiments. Receiver and channel effects are studied.	Solutions to channel and receiver effects are not provided
[75]	Wireless fingerprinting	U	Experiments at various distances are conducted	Solutions to channel effects are not provided
[14]	Wireless fingerprinting	U	Consideration on openset/zero-shot classification	Solutions to channel effects are not provided
[18]	Wireless fingerprinting	U	Large-scale dataset of 100 LoRa devices. Both outdoor and indoor environments	-
[17]	Wireless fingerprinting	U	Design of channel independent spectrogram to mitigate channel effects.	Low SNR outdoor experiments are missing

* (C—Confidentiality, I—Integrity, A—Availability, U—Authentication).

## Data Availability

Not applicable.
